# Bleb morphology and histology in a rabbit model of glaucoma filtration surgery using Ozurdex^®^ or mitomycin-C

**Published:** 2012-03-26

**Authors:** Jeffrey R. SooHoo, Leonard K. Seibold, Ashley E. Laing, Malik Y. Kahook

**Affiliations:** Department of Ophthalmology, University of Colorado Denver, Aurora, CO

## Abstract

**Purpose:**

To determine the effect of a sustained-release dexamethasone implant (Ozurdex^®^) on wound healing after glaucoma filtration surgery in a rabbit model.

**Methods:**

Twelve New Zealand white rabbits were divided into three groups: filtration surgery with intraoperative subconjunctival implantation of Ozurdex^®^ (n=6), filtration surgery with intraoperative topical application of mitomycin-C (MMC; n=6), and filtration surgery with intraoperative topical application of balanced salt solution (BSS; n=12). A standard scale was used to grade bleb vascularity at three and six weeks after the initial operation. Bleb survival was also recorded for comparison between the three groups. Histologic analysis was performed with attention to cellularity and collagen deposition.

**Results:**

MMC-treated blebs demonstrated decreased numbers of goblet cells compared to all other groups. Blebs treated with Ozurdex^®^ maintained a near normal number of goblet cells with modest collagen deposition. The control eyes treated with only BSS had significant collagen deposition and increased cellularity compared to both the Ozurdex^®^ and MMC groups. Bleb vascularity was not significantly different among groups at the three and six week post-operative evaluations. MMC-treated and Ozurdex^®^-treated blebs had significantly prolonged bleb survival compared to blebs treated with only BSS. In addition, MMC-treated blebs had significantly longer survival compared to Ozurdex^®^-treated blebs.

**Conclusions:**

The results of this study support the utility of extended-release dexamethasone (Ozurdex^®^) as a wound modulating agent in a rabbit model of filtration surgery. Further animal and human studies are needed to better characterize a possible role for Ozurdex^®^ in filtration surgery.

## Introduction

Modern glaucoma filtration surgery requires construction of a wound that strikes a balance between under- and over-filtration to achieve adequate long-term control of intraocular pressure. The natural healing processes of the eye favor scar formation after incisional surgery, which can limit bleb function. Modulation of the post-operative healing response after guarded filtration surgery for glaucoma has historically relied on the antifibrotics mitomycin-C (MMC) and 5-fluorouracil (5-FU). These agents have been shown to be effective and their use is supported by large clinical trials and decades of use in human subjects [1-7]. Newer agents targeting specific growth factors, such as anti-vascular endothelial growth factors (anti-VEGF) and anti-transforming growth factor β2 (anti-TGF-β2), have also recently been under investigation [8-11].

Steroids, in particular glucocorticoids, have widespread applications in ophthalmology due to their anti-inflammatory properties. Glucocorticoids act in a nonspecific fashion to blunt immune and inflammatory responses and can influence several components of the wound healing pathway [5,12]. Ultimately, glucocorticoids modulate wound healing by decreasing vascular permeability leading to decreased leukocyte migration and activity in damaged tissues [5]. Use of topical corticosteroids after glaucoma filtration surgery is considered standard of care and has been shown to augment surgical success [13].

Ozurdex^®^ (Allergan, Irvine, CA) is a polymer-based sustained-release intravitreal drug delivery system containing 0.7 mg of the glucocorticoid dexamethasone. Given the known wound modulation properties of glucocorticoids, we hypothesized that Ozurdex^®^ would have a measurable and sustained effect on bleb morphology and histology after filtration surgery. This study compares outcomes in a rabbit model of filtration surgery using MMC, Ozurdex^®^, or balanced salt solution (BSS) control.

## Methods

### Study design

All procedures were performed in accordance with The Association for Research in Vision and Ophthalmology (ARVO) Statement for the Use of Animals in Ophthalmic and Vision Research. The eyes of twelve New Zealand white rabbits were divided into three groups: filtration surgery with intraoperative subconjunctival implantation of Ozurdex^®^ (n=6), filtration surgery with intraoperative topical application of MMC (n=6) and filtration surgery with intraoperative topical application of BSS (n=12). The number of rabbits was chosen based on our experience in previous studies with a similar animal model. No data were available to calculate sample sizes since the medication of interest has not been studied in this fashion to our knowledge. All rabbits underwent glaucoma filtration surgery in the right eye performed by a single surgeon (M.Y.K.) using an established technique described by Cordeiro and colleagues [8]. The left eye of each rabbit acted as the control (n=12) and underwent filtration surgery without the use of an intraoperative wound-modulating agent.

### Glaucoma filtering surgery technique

Each rabbit was anesthetized using a combination of intramuscular ketamine and xylazine (ketamine 40 mg/kg; xylazine 20 mg/kg) and topical anesthesia (2% lidocaine gel) before initiation of surgery. A fornix-based conjunctival dissection was completed and a 23-gauge needle was used to create a scleral tunnel one millimeter posterior to the limbus for insertion of a 22-gauge cannula (Insyte^®^; Becton Dickinson Vascular Access, Sandy, UT) into the anterior chamber. The cannula was inserted such that the distal end crossed the pupillary margin to avoid tube-iris capture. The tube was then secured to the scleral bed with 10–0 nylon suture (Ethicon Inc., Somerville, NJ) and efflux of fluid into the subconjunctival space was confirmed. In the Ozurdex^®^ group, the medication pellet was injected into a sterile plastic cup using the manufacturer’s injecting system. The pellet was then grasped with 0.12 forceps and placed immediately posterior to the proximal end of the implanted cannula. In the MMC group, two 3×3 mm partial-thickness Weck-cel^®^ spears (Alcon Surgical, Fort Worth, TX) soaked in 0.4 mg/ml MMC solution were placed over the scleral bed for 5 min before insertion of the 22-gauge cannula. The scleral bed was then irrigated with 40 ml of BSS after removing the soaked spears and the surgical procedure was completed. The left eye of each animal (in both groups) underwent the exact procedure as noted above for the MMC-treated eyes except the Weck-cel^®^ spears were soaked in BSS instead of MMC. In all eyes, topical moxifloxacin 0.5% solution (Vigamox^®^; Alcon, Fort Worth, TX) and prednisolone acetate 1% suspension (Predforte^®^; Allergan, Irvine, CA) were each instilled four times per day for seven days following surgery.

### Post-operative evaluation

A daily handheld slit lamp examination of the operated eyes following surgery was conducted to document any changes at the surgical site as well as to complete bleb vascularity assessments. Anterior segment photographs were obtained weekly for the duration of the study in each eye. These daily examinations and photographs were evaluated for the presence of implant migration or extrusion. A single, masked independent investigator objectively graded each bleb for survival and vascularity based on slit lamp photography. The primary outcome metric was bleb histology and bleb survival, which was defined as the presence of an elevated subconjunctival fluid pocket at the surgical site. Central bleb vascularity was also graded from photographs taken at three and six weeks post-operatively using a standard scale as noted in Table 1. Intraocular pressure was not collected as an endpoint since our specific metric of interest involved histologic differences between groups.

**Table 1:** Grading of central bleb vascularity.

**Central bleb vascularity score**	**Central bleb vascularity**
0	Avascularity (>50% avascularity)
1	Reduced vascularity (<50% avascularity)
2	Normal vascularity
3	Increased vascularity (<50% increased vascularity)
4	Hypervascularity (>50% increased vascularity)

### Histology

All animals were euthanized at the end of the six-week study. All eyes were then enucleated and immediately immersed in a mixture of 4% paraformaldehyde and 2.5% neutral buffered formalin for 24 h. The globes were dehydrated, embedded in paraffin and sent for microtome sectioning and staining (Hematoxylin and Eosin and Masson Trichrome; Sigma, St. Louis, MO). A modified semi-quantitative grading system to assess cellularity and collagen deposition was used to compare findings between the three groups (Table 2) [14]. Goblet cell number was calculated using the average cell number per high-powered field from six consecutive central bleb cross-sections of each specimen (Table 2). A prior study has established that normal rabbit conjunctiva typically has about seven goblet cells per high-powered field [15]. A masked evaluation was then performed on all samples. Statistical analysis using one-way analysis of variance (ANOVA) test was completed for all data sets. A p-value <0.05 was considered statistically significant.

**Table 2:** Bleb histology.

**Group**	**Collagen deposition (Masson trichrome)**	**Cellularity (H&E)**	**Mean goblet cell number (SD)**
Mitomycin C	±	±	1.33 (0.82)
Ozurdex®	++	+	6.83 (0.75)
BSS control	+++	+++	7.33 (1.03)

-=absent; ±=weakly present; +,++,+++=present. H&E=Hematoxylin and Eosin. SD=Standard Deviation. p<0.001 for the comparison of goblet cell number in eyes treated with MMC versus those treated with Ozurdex® or BSS. p=0.31 for the comparison of goblet cell number in eyes treated with Ozurdex® versus those treated with BSS.

## Results

**Figure 1:**
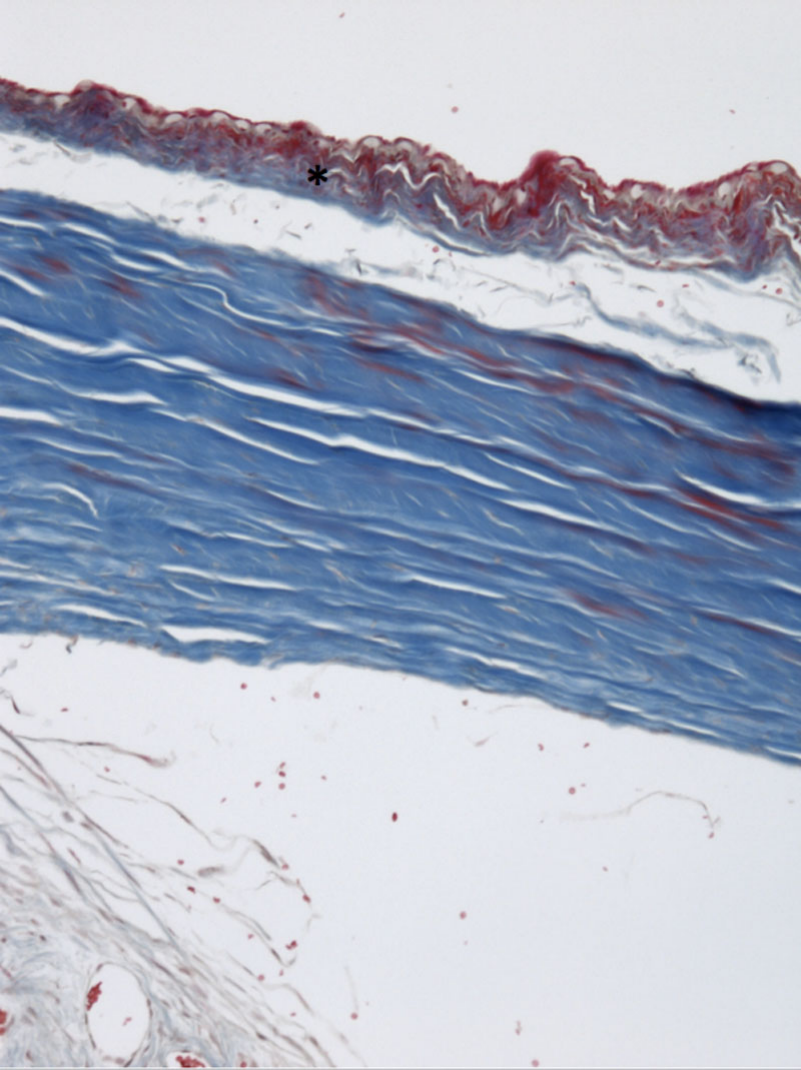
MMC-treated tissue was noted to have less collagen deposition (asterisk) with Masson Trichrome stain compared to Ozurdex^®^- or BSS-treated eyes as well as loss of normal distribution of goblet cells in all sections (10× magnification).

**Figure 2:**
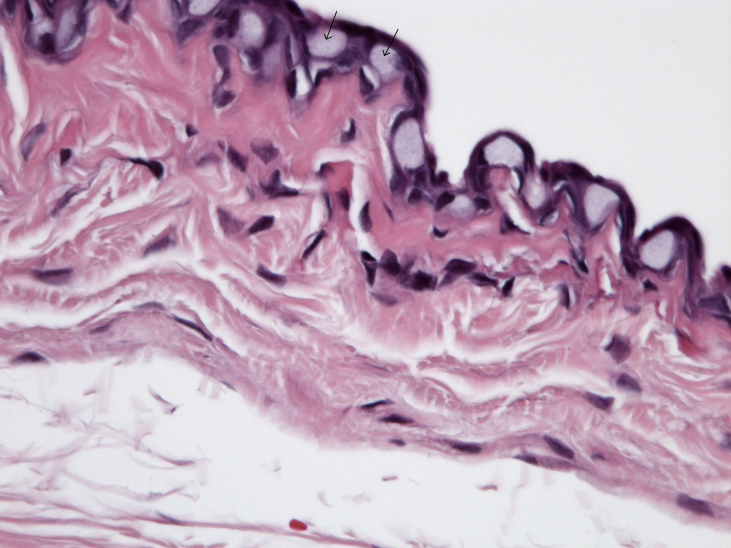
A normal number of conjunctival goblet cells (arrows) per high power field with Hematoxylin and Eosin stain was noted in sections from the Ozurdex^®^-treated eyes (40× magnification).

**Figure 3:**
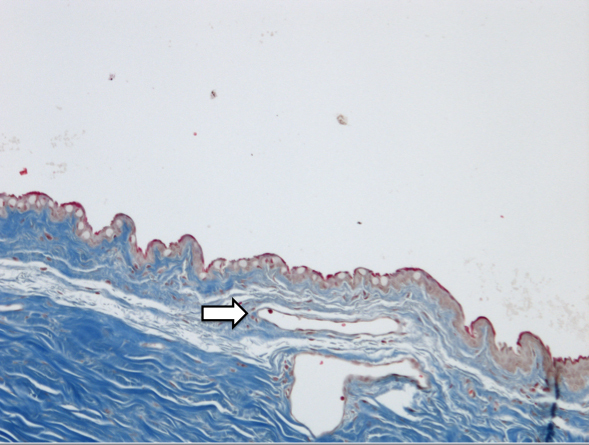
Histologic analysis of bleb tissue revealed less collagen deposition with Masson Trichrome stain compared to the BSS control as well as a thin capsule (white arrow) at the location of the Ozurdex^®^ implant (10× magnification).

**Figure 4:**
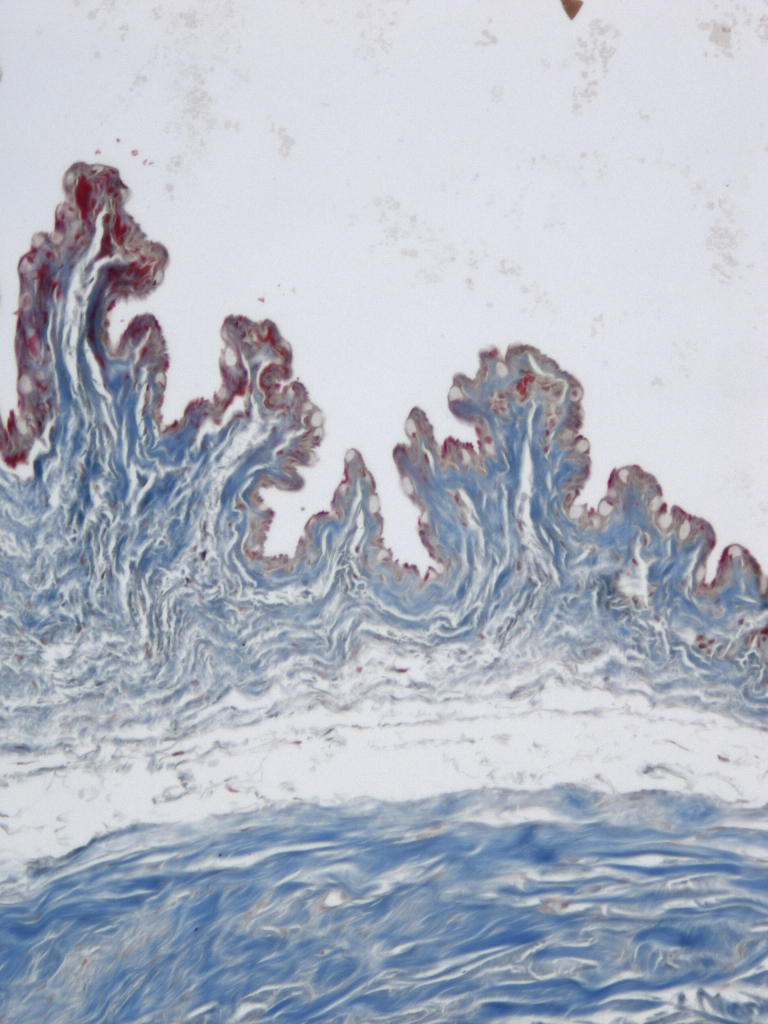
Exuberant collagen deposition with Masson Trichrome stain was noted in all evaluated sections from BSS-treated controls (10× magnification).

Blebs treated with MMC demonstrated a decrease in number of goblet cells along with minimal collagen deposition and cellularity compared to all other eyes (p<0.001; Figure 1 and Table 2). In contrast, Ozurdex^®^-treated blebs maintained a normal number of goblet cells compared to BSS control (p=0.31; Figure 2) with modest collagen deposition and increased cellularity as shown in Table 2. There was a thin capsule surrounding the location of all Ozurdex^®^ implants but no evidence of foreign body reaction (Figure 3). The BSS-treated eyes had significant collagen deposition and increased cellularity compared to both the Ozurdex^®^ and MMC groups (Figure 4 and Table 2). In addition, the BSS-treated eyes maintained a normal number of goblet cells compared to previously published literature [15]. Bleb vascularity was similar between groups at both three and six weeks follow up (p>0.05 for all comparisons; Table 3).

**Table 3:** Mean post-operative vascularity of all groups after three and six weeks of follow-up.

**Group**	**Three weeks (SD)**	**Six weeks (SD)**
Mitomycin-C (n=6)	1.17 (1.47)	1.50 (1.64)
Ozurdex® (n=6)	1.83 (1.47)	2.00 (1.67)
Control (n=12)	2.08 (1.16)	2.50 (1.38)

SD=Standard Deviation. p>0.05 for comparison of bleb vascularity between all groups.

Table 4 lists post-operative bleb survival for each of the three groups. MMC-treated and Ozurdex^®^-treated blebs demonstrated a statistically significant increase in duration of post-operative bleb survival compared to the BSS control group (p<0.001 for both comparisons). There was also a significant difference in bleb survival in the comparison between the MMC and Ozurdex^®^ groups in favor of MMC-treated eyes (p=0.001).

**Table 4:** Post-operative bleb survival.

**Group**	**Bleb Survival – Days (SD)**	**Range**
Mitomycin-C (n=6)	39.33 (3.56)	33–42
Ozurdex® (n=6)	30.17 (3.43)	26–35
Control (n=12)	11.17 (3.27)	5–16

SD=Standard Deviation. p<0.001 for comparison of bleb survival in eyes treated with BSS versus those treated with Ozurdex® or MMC. p=0.001 for comparison of bleb survival in eyes treated with Ozurdex® versus those treated with MMC (in favor of MMC-treated eyes).

There were no documented cases of implant extrusion, endophthalmitis, corneal epithelial toxicity, or persistent anterior chamber inflammation in all treated eyes.

## Discussion

Our findings support the utility of extended-release dexamethasone for wound modulation in a rabbit model of filtration surgery. The results of this study documented that use of Ozurdex^®^ resulted in improved bleb survival compared to BSS control and favorable bleb histology compared to the MMC group. Ozurdex^®^ is currently approved by the United States Food and Drug Administration for the treatment of macular edema following retinal vein occlusion as well as noninfectious posterior uveitis. In the current study, Ozurdex^®^-treated eyes demonstrated favorable bleb morphology and histology. The stable density of goblet cells combined with modest fibroblast proliferation and collagen deposition suggest that the use of sustained-release steroid may allow for the formation of thicker, more stable blebs. It remains unclear what the impact might be on the degree of intraocular pressure lowering in this scenario. The Ozurdex^®^ and MMC groups both demonstrated a significantly prolonged bleb survival time compared to controls. The significant difference between the Ozurdex^®^ and MMC groups in bleb survival might be predictable, but the increased survival of Ozurdex^®^ blebs over that of BSS-treated blebs suggests potential for sustained-release dexamethasone for wound modulation after filtration surgery.

The process of wound healing is complex and involves multiple vascular and cellular pathways with cross interactions that are not fully understood [5]. While modern glaucoma filtration surgery with concurrent use of 5-FU or MMC is a well recognized and successful technique, the opportunity exists to explore the use of other agents to augment surgical success while at the same time decreasing the risk of post-operative complications. Mitomycin-C acts as an antiproliferative agent via DNA cross-linking, which in turn inhibits both DNA replication and protein synthesis [5]. With regards to glaucoma filtration surgery, these inhibitory effects decrease fibroblast proliferation leading to decreased scar formation and improved bleb function and survival [16-18]. Use of MMC, however, does increase the risk of postoperative bleb leak, hypotony, and endophthalmitis [5,19,20]. Due to the multifactorial nature of wound healing, it is likely that multiple agents, used either concurrently or sequentially, may be needed to achieve the aforementioned goals [21]. The exact combination, dose, and route of administration of treatments that produces the best surgical outcome with minimal side effects is not yet determined and is a worthwhile area of research.

The efficacy of topical post-operative medications such as corticosteroids is by nature dependent on a patient’s ability to properly instill the medications while following the appropriate timeline for their use. Intraoperative retrobulbar triamcinolone acetonide has been shown to augment the success of trabeculectomy with MMC, but the duration of action is short and instillation is difficult due to reflux of fluid from the surgical site [22]. Others have reported success with injection of triamcinolone acetonide directly into the bleb at the conclusion of surgery [23].

This animal model provides insight regarding the ideal regimen for wound modulation following filtration surgery but does have significant limitations. The size of the treatment groups in this pilot study was small, although in line with previously published studies investigating bleb characteristics in rabbits, and it remains to be seen what effect sustained-release corticosteroids may have on long-term bleb function and survival using a larger number of animals. It is also noted that results from an animal study may not be directly applicable to human patients. The rabbit is, however, an established model for the evaluation of novel techniques in glaucoma filtration surgery [24-26].

A sustained-release corticosteroid implant such as Ozurdex^®^ does have many possible advantages. However, its use for filtration surgery is limited due to its original design for intravitreal use. Enhancements could include a wider and flatter profile that would lessen the chance of conjunctival erosion and increase the amount of tissue exposed to medication as well as tailoring the dose and time release of active ingredient specifically for wound modulation. Unlike the use of MMC, it is not possible to vary the concentration, treatment area, or duration of exposure to Ozurdex^®^. Cost and availability of Ozurdex^®^ may also limit its use in resource-poor treatment areas. As always, the ophthalmic side effects of corticosteroids, including cataract formation and increased intraocular pressure, need to be carefully weighed against the benefits of therapy.

The results of this preliminary rabbit study suggest a role for Ozurdex^®^ as a wound modulator after traditional glaucoma filtration surgery. Compared to blebs treated with MMC, Ozurdex^®^-treated blebs were thicker with less disruption of baseline cellular architecture. Use of Ozurdex^®^ could potentially allow for combination therapy with MMC or 5-FU. For example, using a lower dose of MMC with Ozurdex^®^ might lead to improved wound modulation while also decreasing the incidence of MMC-associated complications. The use of a sustained drug delivery system may also allow for more consistent and reproducible results compared to the variability of compounding pharmacy MMC doses as well as the variable techniques used by surgeons when applying MMC intra-operatively. Further studies are needed to better characterize and expand on these preliminary results and human studies would be needed to fully understand the surgical and clinical utility of this wound modulation approach.
